# Machine learning-driven prediction models and mechanistic insights into cardiovascular diseases: deciphering the environmental endocrine disruptors nexus

**DOI:** 10.1186/s12967-025-07223-6

**Published:** 2025-11-12

**Authors:** Wen-Min Yu, Yu-Peng Chen, An-Le Cheng, Zi-Yi Zheng, Jia-Wen Wang, Xiong-Bo Liu, Jing-Xuan Zhou

**Affiliations:** 1 Department of Cardiology, Jintan Affiliated Hospital of Jiangsu University, Changzhou, China; 2 Department of Urology, Shengli Clinical Medical College of Fujian Medical University, Fujian Provincial Hospital, Fuzhou, China; 3 Department of Urology, Xiamen Hospital of Traditional Chinese Medicine, Xiamen, China; 4 School of Urban Planning and Design, Peking University Shenzhen Graduate School, Shenzhen, China; 5 Department of Urology, Fujian Provincial Hospital, Fuzhou University Affiliated Provincial Hospital, Shengli Clinical Medical College of Fujian Medical University, Fuzhou, China

**Keywords:** Endocrine-disrupting chemicals, Cardiovascular disease, Oxidative stress, Molecular dynamics simulations

## Abstract

**Background:**

Cardiovascular disease (CVD) persists as the foremost cause of global mortality, yet the mechanistic links between environmental pollutants and CVD pathogenesis remain poorly defined. This study addresses this gap by integrating machine learning-driven epidemiology with computational biology to systematically evaluate the role of endocrine-disrupting chemicals (EDCs) in CVD development.

**Method:**

We analyzed data from the NHANES cohort to identify CVD-associated EDCs using advanced predictive modeling. Molecular docking and dynamics simulations were employed to characterize interactions between prioritized compounds and the NOX2-p22phox complex, a key regulator of oxidative stress. Structural and functional impacts on NADPH oxidase activity were assessed through residue-level binding analysis and reactive oxygen species (ROS) quantification.

**Results:**

Machine learning identified 3-hydroxyfluorene (3-HF) as a novel environmental risk factor for CVD. Molecular simulations revealed that 3-HF selectively binds to the transmembrane domain of the NOX2-p22phox complex, forming stable interactions with residues critical for structural integrity (e.g. T135, H160). These interactions destabilized the protein complex, impairing NADPH oxidase assembly and suppressing ROS generation. Further analysis demonstrated that 3-HF-mediated oxidative stress disruption correlates with vascular dysfunction pathways implicated in CVD progression.

**Conclusion:**

This study establishes 3-HF as a redox-disrupting environmental contaminant contributing to CVD through NOX2-p22phox targeting. By bridging population-level exposure data with atomic-scale mechanistic insights, our work provides a transformative framework for environmental health risk assessment and preventive intervention design.

**Supplementary Information:**

The online version contains supplementary material available at 10.1186/s12967-025-07223-6.

## Introduction

Cardiovascular disease (CVD) is the leading cause of death and disability worldwide, accounting for approximately 32% of all deaths. This complex group of diseases includes coronary heart disease, hypertension, heart failure, stroke, and peripheral arterial disease. Its development is linked to endothelial dysfunction, lipid metabolism disorders, oxidative stress, and chronic inflammation [1, 2]. While lifestyle, genetics, smoking, alcohol consumption, and aging are well-established risk factors for CVD [3], they do not fully explain its prevalence trends or individual susceptibility [4]. In recent years, environmental pollutants have emerged as a significant yet often overlooked contributor to the rising burden of CVD. However, their mechanisms of action remain inadequately understood.

Epidemiological studies suggest that long-term exposure to environmental endocrine disruptors chemicals (EDCs) may increase the risk of CVD [5]. EDCs, including phenolic compounds, phthalates, and polycyclic aromatic hydrocarbons (PAHs), are widely present in air, water, food packaging, and personal care products [6]. These pollutants can bioaccumulate, disrupt hormone signaling pathways, and contribute to CVD through oxidative stress and inflammation [7–9]. However, research on the combined effects of multiple environmental chemicals on CVD risk remains limited. Traditional statistical methods struggle to analyze these complex exposure-health relationships, making it difficult to capture nonlinear effects and interactions [10].

Machine learning (ML) has gained traction in biomedical and epidemiological research due to its ability to handle high-dimensional data, model complex exposure-response relationships, and integrate multi-source information [11, 12]. Despite its potential in disease prediction, ML-based studies systematically assessing the impact of combined EDCs exposure on CVD risk remain scarce, particularly in quantifying additional effects beyond traditional risk factors.

Alongside epidemiological approaches, computational biology has emerged as a valuable tool in environmental health research. It enables the prediction of interactions between EDCs and key cardiovascular targets [13], while assessing binding stability and potential biological effects. Integrating computational biology with epidemiological data offers deeper insights into the molecular mechanisms linking environmental pollutants to CVD, informing evidence-based environmental health policies [14]. However, few studies have systematically combined ML and computational biology to explore EDCs exposure and CVD risk, highlighting a critical research gap.

This study utilizes data from the National Health and Nutrition Examination Survey (NHANES, 2003–2016) to evaluate the association between EDCs exposure and CVD using ML models. By constructing multiple predictive models, it quantifies the effects of combined exposure to phenolic compounds, phthalates, and PAHs on CVD risk. SHAP analysis is employed to identify key environmental pollutants and their independent contributions. Additionally, molecular docking and molecular dynamics simulations predict interactions between high-risk pollutants and key cardiovascular targets, uncovering potential toxicological effects. The findings will provide new epidemiological evidence on the impact of environmental pollutants on CVD and support precision prevention and environmental intervention strategies.

## Methodology

**Fig. 1:**
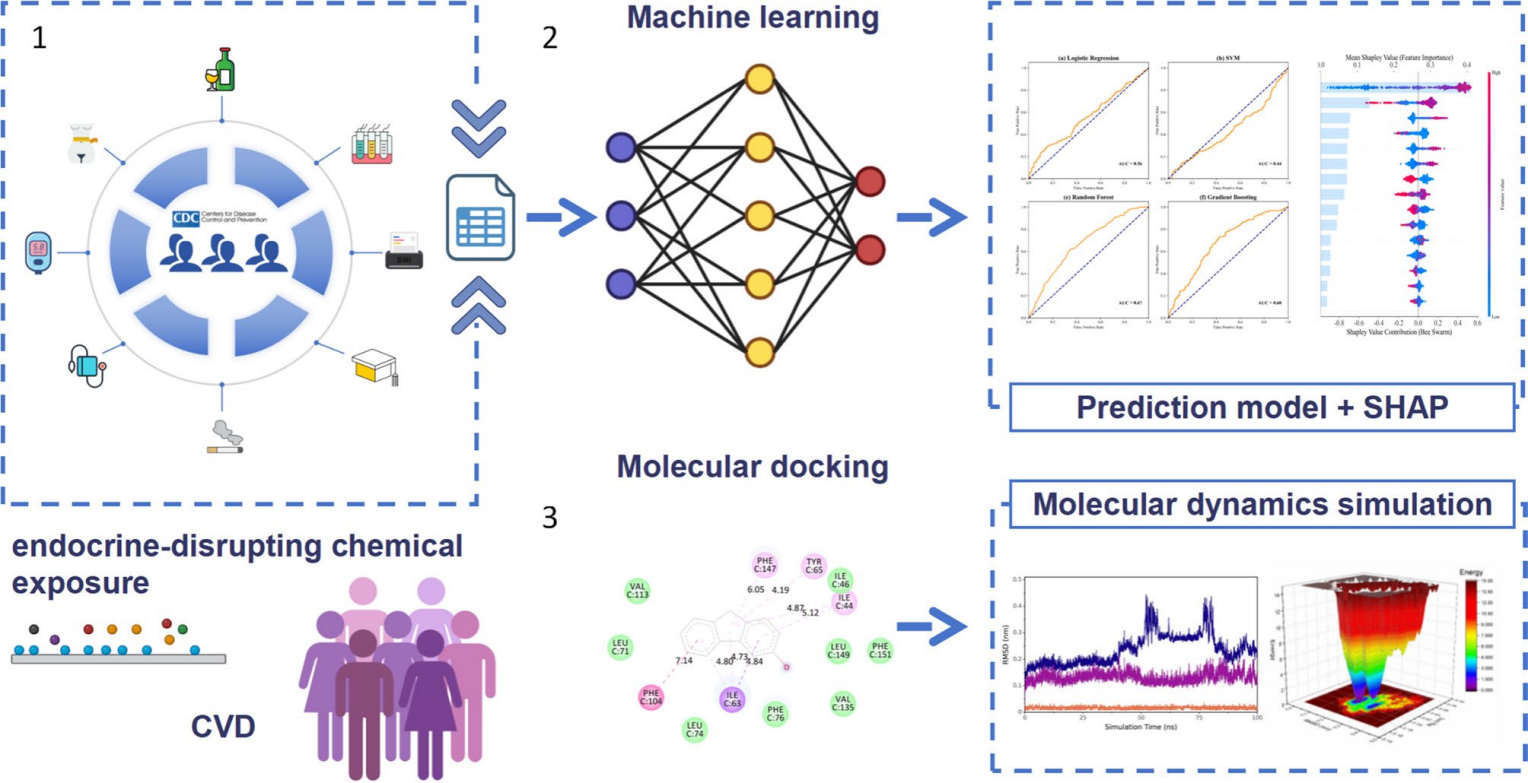
Overall flowchart of the study

The overall flow chart of the study is shown in Fig. 1.

### Study population

**Fig. 2:**
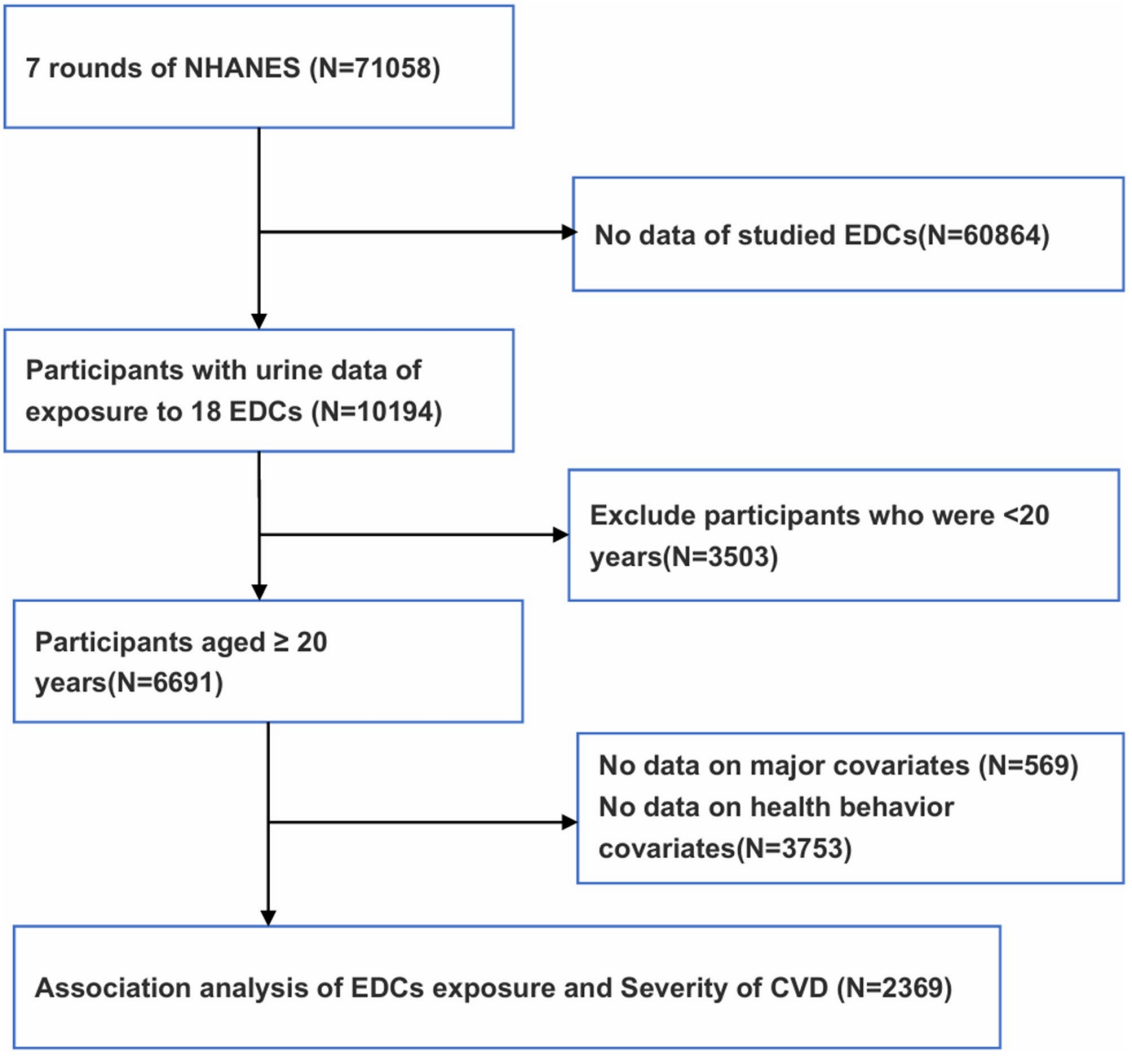
Flowchart of the participants included in the final analysis (*N* = 2,369), NHANES, USA, 2003–2016

This study examined urinary concentrations of previously studied EDCs, including phenols, phthalates, and PAHs. A total of 71,058 participants from seven consecutive NHANES cycles (2003–2016) were included, as phenol data were unavailable in earlier cycles. Through cross-cycle data intersection, we identified 18 target compounds: three phenols, nine phthalates, and six PAHs metabolites. From an initial pool of 10,194 participants, exclusions were made for those under 20 years old (n = 3,503), those missing major covariates (n = 569), and those lacking health behavior data (n = 3,753), resulting in a final sample of 2,369 eligible participants for analysis (Fig. 2).

### Evaluation of the severity of CVD

This study assessed CVD risk based on five established risk factors identified by the American Heart Association: hypertension, hypercholesterolemia, obesity, diabetes mellitus, and smoking. Each factor was coded as a dichotomous variable (1 = present, 0 = absent) with the following thresholds: diastolic-systolic blood pressure (DBP-SBP) ≥80/130 mmHg, total cholesterol ≥240 mg/dL, body mass index (BMI) ≥30 kg/m^2^, and fasting blood glucose ≥126 mg/dL. Smoking status was determined based on questionnaire responses, with individuals classified as smokers if they had a lifetime consumption of more than 100 cigarettes. A cumulative CVD risk point (range: 0–5) was calculated for each participant by summing these factors [15, 16]. To classify CVD risk, participants were grouped into low or no risk ( < 2 points) and high risk (≥2 points). This binary classification was then used to examine the association between EDCs exposure and CVD risk [15].

### Measurements of EDCs in urine

Urine specimens were obtained during mobile examination center (MEC) visits and cryopreserved at − 20 °C prior to batch transportation to the National Center for Environmental Health for chemical quantification. This study quantified 18 EDCs and their metabolic derivatives, categorized into four classes: (1) phenolic compounds (bisphenol A [BPA], triclosan [TCS], benzophenone-3 [BP-3]); (2) nine phthalate metabolites (MECPP, MnBP, MCPP, MEP, MEHHP, MEHP, MiBP, MEOHP, MBzP); and (3) six PAHs biomarkers (1-Hydroxynapthol, 2-Hydroxynapthol, 2-Hydroxyfluorene, 3-Hydroxyfluorene, 1-Hydroxyphenanthrene, 1-Hydroxypyrene). To account for urinary dilution variability, all measured concentrations were normalized to creatinine levels following established protocols [17, 18].

### Covariates

Covariates specifically selected from questionnaire data on demographics, socio-economic status, health behaviors and health status [19–21]. This study used age, sex, race/ethnicity (Mexican American, other Hispanic origins, non-Hispanic White, non-Hispanic Black, and other race), PIR level, education level (under high school, high school or equivalent, and above high school), marital status as covariates.

### Modeling

Statistical analyses followed NHANES complex survey design protocols, incorporating sample weights, stratification variables, and primary sampling units per official analytical guidelines. The analysis included 24 input variables: 19 continuous measures (18 urinary EDCs concentrations and age) and five categorical factors (race, education level, sex, marital status, and poverty-income ratio [PIR]) [22]. The dataset was split into training (70%, n = 1,658) and testing (30%, n = 711) subsets.

Twelve machine learning (ML) models were applied to assess the association between EDCs exposure and cardiovascular disease (CVD), including Logistic Regression (LR), Support Vector Machine (SVM), K-Nearest Neighbors (KNN), Decision Tree (DT), Random Forest (RF), Gradient Boosting (GB), Extreme Gradient Boosting (XGBoost), Light Gradient Boosting Machine (LightGBM), CatBoost, Naïve Bayes, Multi-Layer Perceptron (MLP), and AdaBoost [18, 23]. To improve model performance, urinary EDC concentrations were log-transformed [18, 24].

Model reliability was evaluated using standard performance metrics. Discrimination ability was assessed via the area under the receiver operating characteristic curve (AUC), while additional metrics—including sensitivity (recall), specificity, average precision (AP), false positive rate (FPR), false discovery rate (FDR), positive predictive value (PPV), negative predictive value (NPV), and the F1 score—were used to ensure a balanced assessment of precision and recall. To mitigate overfitting and validate model robustness, both five-fold and 10-fold cross-validation were performed within the training cohort, ensuring the generalizability and stability of predictive outcomes.

To enhance interpretability and address the “black-box” nature of ML models, SHapley Additive Explanations (SHAP) was used to quantify feature importance. In our analysis, all 24 variables were initially included for model training. To improve interpretability, we applied SHAP values to rank variable importance and primarily reported the top 15 predictors. This procedure effectively reduced redundancy among highly correlated variables. Although multicollinearity may still exist in the full set of predictors, the combined use of robust algorithms (CatBoost) and SHAP-based feature ranking mitigated its potential adverse impact on model performance and interpretation. This approach has also been applied and validated in related cardiovascular disease studies, supporting its effectiveness in enhancing model robustness, stability, and interpretability [25].

### Molecular docking

We performed molecular docking analysis using four CVD-related proteins (PDB IDs: 3FXI, 2HEU, 3DZU, and 4YAY) and the highest-weighted EDC selected by machine learning. 3FXI activates the NF-κB pathway, promoting atherosclerotic plaque formation [26]; 2HEU induces the production of reactive oxygen species (ROS) and causes endothelial dysfunction [27]; 3DZU is responsible for regulating adiponectin secretion and inhibiting the formation of foam cells [28]; 4YAY can mediate vascular smooth muscle contraction and promote hypertension [29]. Protein structures were optimized in multiple phases via the GROMACS 2018 (GROMOS 54a7 force field) for multi-stage optimization [30]. Ligands were prepared in ChemOffice 22.0 with the MMFF94 force field, generating 500 low-energy conformations per compound, followed by Gasteiger-Marsili charge calculations and RMSD validation. Docking was performed using the Lamarckian genetic algorithm (LGA) in CavityPlus, with binding pockets predefined to identify the optimal conformation [31]. Hydrogen bonding and hydrophobic interactions were analyzed using PyMol 1.3, and structural outputs were converted to PDB format via OpenBabel 2.4.0. All parameters, including force field definitions, optimization steps, and scoring functions, were validated against co-crystal experimental data and Schrödinger plasma calculations. The best binding conformation was determined based on the highest docking score.

### Molecular dynamics simulation

Molecular dynamics (MD) simulations were conducted using the Gromacs2022 program. The GAFF force field was used for small molecules, the AMBER14SB force field for proteins, and the TIP3P water model for solvation. Protein and small molecule ligand files were merged to construct the complex simulation system, which was run under periodic boundary conditions at constant temperature and pressure. During the MD simulations, all hydrogen bonds were constrained using the LINCS algorithm with a 2 fs integration step. Electrostatic interactions were calculated using the Particle-Mesh Ewald (PME) method, with a cut-off value of 1.2 nm, while non-bonded interactions had a cut-off of 10 Å, updated every 10 steps. The temperature was maintained at 298 K using the V-rescale temperature coupling method, and pressure was regulated at 1 bar using the Berendsen method. NVT and NPT equilibrium simulations were performed for 100 ps each at 298 K. A 100 ns MD simulation was carried out for the complex system, saving the conformation every 10 ps. After completing the simulations, the trajectories were analyzed using VMD and PyMOL, and the free energy of MMPBSA binding between the protein and the small molecule ligand was calculated using the g_mmpbsa program.

## Results and discussion

### Descriptive statistics

A total of 2,369 participants were included in the final analysis, selected from an initial pool of 71,058 screened candidates. The mean age of the study population was 48.4 years, with males accounting for 50.8% of the participants, including 939 cases of CVD. Ethnic distribution was predominantly non-Hispanic White (47.5%), followed by non-Hispanic Black (19.2%) and Mexican American (15.4%) participants. In terms of education, 50.9% of participants had attained a college-level education. Regarding lifestyle factors, 45.4% of participants reported tobacco use. A detailed comparison of demographic and clinical characteristics between the CVD-affected and unaffected groups is provided in Table 1.

**Table 1:** Characteristics of participants grouped by risk level

Variables	Overall(n = 2369)	High-risk CVD Participatants(n = 949)	Low-risk CVD Participatants(n = 1430)	P
**Age,years**	48.4 ± 17.6	53.7 ± 16.0	45.0 ± 17.8	< 0.001
**Sex,male%**	1203(50.8%)	1334(56.3%)	1251(52.8%)	< 0.001
**Education level, %**				< 0.001
Less than 9th grade	10.80%	10.90%	10.80%	
9-11th grade (Includes 12th grade with no diploma)	14.90%	17.70%	13.10%	
High school graduate/GED or equivalent	23.30%	18.50%	21.70%	
Some college or AA degree	27.50%	27.20%	27.80%	
College graduate or above	23.40%	25.70%	26.60%	
Refused	0.10%	0.10%	0.10%	
**Ethnicity, %**				< 0.001
Mexican American	15.40%	13.00%	16.50%	
Other Hispanic	9.50%	7.80%	10.80%	
Non-Hispanic White	47.50%	51.20%	45.00%	
Non-Hispanic Black	19.20%	23.20%	17.00%	
Other Race	8.40%	4.80%	10.70%	
**PIR**	2.6 ± 1.6	2.5 ± 1.6	2.6 ± 1.6	0.032
**smoker, %**	45.40%	71.35%	28.39%	< 0.001
**LBXTC, mg/dL**	194.1 ± 40.6	205.7 ± 46.4	186.5 ± 34.1	< 0.001
**SBP, mmHg**	122.4 ± 17.6	131.9 ± 18.4	116.2 ± 13.9	< 0.001
**DBP, mmHg**	68.9 ± 12.5	73.0 ± 14.2	66.2 ± 10.5	< 0.001
**GLU, mg/dL**	107.0 ± 32.9	114.4 ± 40.0	102.2 ± 26.1	< 0.001
**BMI, kg/m2**	28.9 ± 6.5	32.1 ± 6.9	26.8 ± 5.3	< 0.001
**MnBP, ng/L**	26.7 ± 88.1	27.1 ± 108.8	26.4 ± 71.3	0.633
**MEP, ng/L**	315.3 ± 1080.1	395.8 ± 1463.3	262.4 ± 721.6	0.07
**MEHP, ng/L**	5.4 ± 24.7	4.4 ± 18.3	6.1 ± 28.1	0.002
**MBzP, ng/L**	12.1 ± 22.3	11.3 ± 17.1	12.7 ± 25.1	0.56
**MCPP, ng/L**	7.6 ± 40.5	7.0 ± 31.8	8.0 ± 45.3	0.152
**MEHHP, ng/L**	44.8 ± 234.3	42.6 ± 166.0	46.2 ± 270.0	0.538
**MEOHP, ng/L**	26.1 ± 145.5	23.9 ± 89.5	27.6 ± 172.7	0.703
**MiBP, ng/L**	12.0 ± 17.3	11.8 ± 17.2	12.1 ± 17.3	0.217
**MECCP, ng/L**	62.4 ± 360.8	55.1 ± 180.0	67.2 ± 440.8	0.755
**BPA, ng/L**	3.6 ± 21.1	3.9 ± 31.6	3.4 ± 9.0	0.534
**BP-3, ng/L**	280.8 ± 1330.6	205.2 ± 1236.0	330.5 ± 1387.4	< 0.001
**TCS, ng/L**	111.7 ± 293.3	95.1 ± 268.9	122.6 ± 307.9	0.08
**1-napthol, ng/L**	52101.2 ± 787968.9	87710.7 ± 1103050.0	28718.4 ± 478380.7	< 0.001
**2-napthol, ng/L**	8447.1 ± 11756.3	9564.6 ± 12605.2	7713.2 ± 11107.4	< 0.001
**3-fluorene, ng/L**	327.8 ± 661.8	404.4 ± 730.4	277.4 ± 607.7	< 0.001
**2-fluorene, ng/L**	636.6 ± 1140.4	796.0 ± 1383.5	532.0 ± 933.2	< 0.001
**1-phenanthrene, ng/L**	219.1 ± 366.8	229.0 ± 363.7	212.6 ± 368.8	0.018
**1-pyrene, ng/L**	217.3 ± 440.8	224.6 ± 392.5	212.5 ± 469.9	0.027

The value before parentheses is the average (continuous) or number (categorical) of the corresponding variable; The numbers in parentheses represent the standard deviation (continuous) or proportion (categorical) of the corresponding variable; P is the level of significance

**Fig. 3:**
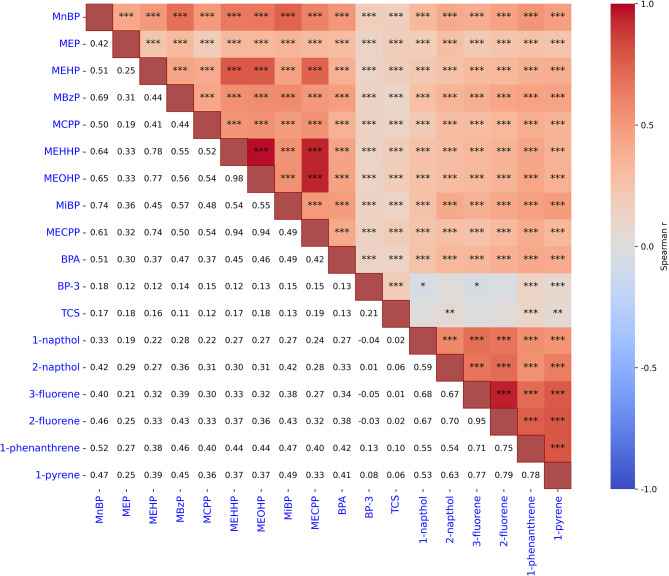
Mutual correlations of the urinary levels of 18 studied EDCs using the Spearman correlation method. The black numbers in the lower left part were the correlation coefficients. The upper right part was the heat map of the correlation coefficients between EDCs concentrations. The white represents a null correlation, the blue represents a positive correlation, and the red represents a negative correlation. The darker the color is, the greater the correlation coefficient is. The black *, **, and *** indicate P < 0.05, P < 0.01, and P < 0.001, respectively

The pairwise correlation between urinary EDCs was analyzed and is shown in Fig. 3. Significant correlations were found between some EDCs, with pairwise correlations ranging from 0.01 to 0.98. The strongest correlation was observed between the urinary levels of MEOHP and MEHHP (r = 0.98, P < 0.001). This strong correlation may result from three interrelated environmental and metabolic drivers. First, these compounds, as major metabolites of di(2-ethylhexyl) phthalate (DEHP), share the same source of exposure, typically precipitating from plasticized consumer products, leading to parallel intake patterns in humans. Second, they undergo enzymatic hydrolysis via the same hepatic pathway, resulting in co-dependent metabolic kinetics that synchronize urinary excretion rates. Third, environmental persistence allows for simultaneous intake via the dietary route, further amplifying the strength of the correlation.

### Model performance comparison

Among the 12 machine learning models evaluated, the CatBoost algorithm demonstrated the highest discriminative efficacy. It achieved optimal clinical applicability for cardiovascular risk stratification by balancing accuracy, recall, NPV, F1 score, and AP (Table 2). Experimental validation confirmed the algorithm’s robustness, as it maintained predictive stability even under noise interference.

**Table 2:** Comparison of model key indicators

Model	Accuracy	S/R	Specificity	Negative Predictive Value (NPV)	False Positive Rate (FPR)	False Discovery Rate (FDR)	F1 Score	Average Precision (AP)
Logistic Regression	0.767	0.976	0.038	0.313	0.962	0.220	0.867	0.780
KNN	0.777	1.000	0.000	NaN	1.000	0.223	0.875	0.777
SVM	0.762	0.939	0.144	0.404	0.856	0.207	0.860	0.792
Random Forest	0.725	0.820	0.394	0.385	0.606	0.175	0.823	0.817
Naive Bayes	0.774	0.944	0.182	0.480	0.818	0.199	0.867	0.800
Decision Tree	0.784	0.928	0.280	0.529	0.720	0.182	0.870	0.815
XGBoost	0.784	0.922	0.303	0.526	0.697	0.178	0.869	0.819
AdaBoost	0.784	0.920	0.311	0.526	0.689	0.177	0.869	0.820
CatBoost	0.782	0.933	0.258	0.523	0.742	0.186	0.870	0.812
LightGBM	0.383	0.243	0.871	0.248	0.129	0.132	0.380	0.799
MLP	0.730	0.848	0.318	0.375	0.682	0.187	0.830	0.808
Ridge Classifier	0.788	0.896	0.409	0.529	0.591	0.159	0.868	0.835

S/R: Sensitivity/Recall

**Fig. 4:**
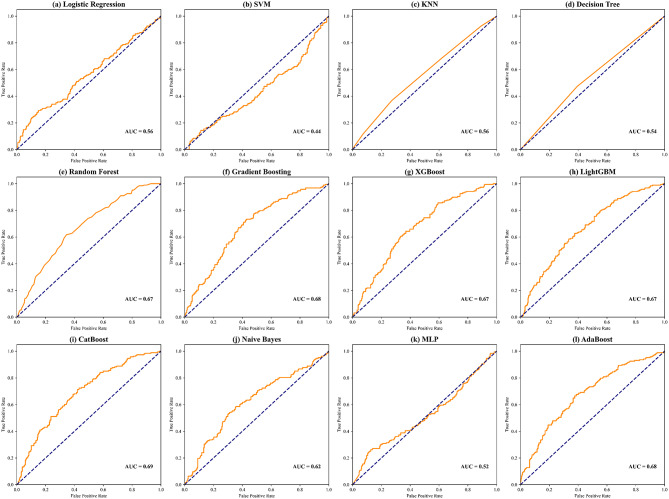
The area under the curve (AUC) of the proposed 12 models (from (**a**) to (**l**)) for overall performance evaluation

ROC curves for all models are presented in Fig. 4. The Ridge Classifier achieved the highest AUC value of 0.7875, though it had a high FPR of 0.591. AdaBoost, XGBoost, and Decision Tree also exhibited AUC values of 0.7841, but underperformed in sensitivity (recall) and NPV. These models were found to be highly sensitive to parameter adjustments, requiring precise fine-tuning for optimal performance. KNN, Naive Bayes, Logistic Regression, SVM, MLP, and RF had relatively lower performance, with AUC values of 0.7774, 0.7740, 0.7673, 0.7622, 0.7302, and 0.7251, respectively, indicating their limited ability to effectively handle the task. LightGBM showed the poorest performance, with an AUC value of only 0.383. This may be attributed to its leaf-wise growth strategy, which overly focuses on local gradient optimization and amplifies bias in cases of insufficient sample size or strong noise, leading to overfitting. Additionally, its histogram-based feature discretization may overlook key threshold information, especially in continuous variable scenarios, diminishing its ability to capture nonlinear target variables.

**Fig. 5:**
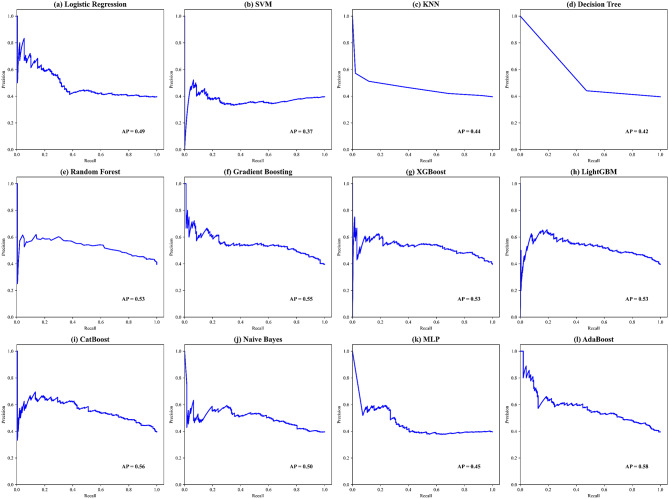
The precision-recall curve of the proposed 12 models (from (a) to (l)) for handling imbalanced data performance evaluation

The AUC and precision-recall curves for the 12 machine learning models are presented, with CatBoost demonstrating the strongest AUC performance and highest recall among all models. CatBoost reduces overfitting through its automatic and efficient processing of categorical features, ordered boosting strategy, and symmetric tree structure optimization, which helps to mitigate gradient bias. These features allow the model to more accurately capture complex data patterns, making it particularly well-suited for large-scale and multivariate datasets (Fig. 5). Additionally, the confusion matrix of the 12 ML models’ performance is displayed, showcasing their respective capabilities [32] (Figure S1). A comprehensive feature-based analysis confirms that CatBoost achieves the highest accuracy and resilience in identifying CVD risk scores. Furthermore, CatBoost excels in improving training speed and model generalization, making it the ideal choice for addressing such challenges.

### Model explanation

**Fig. 6:**
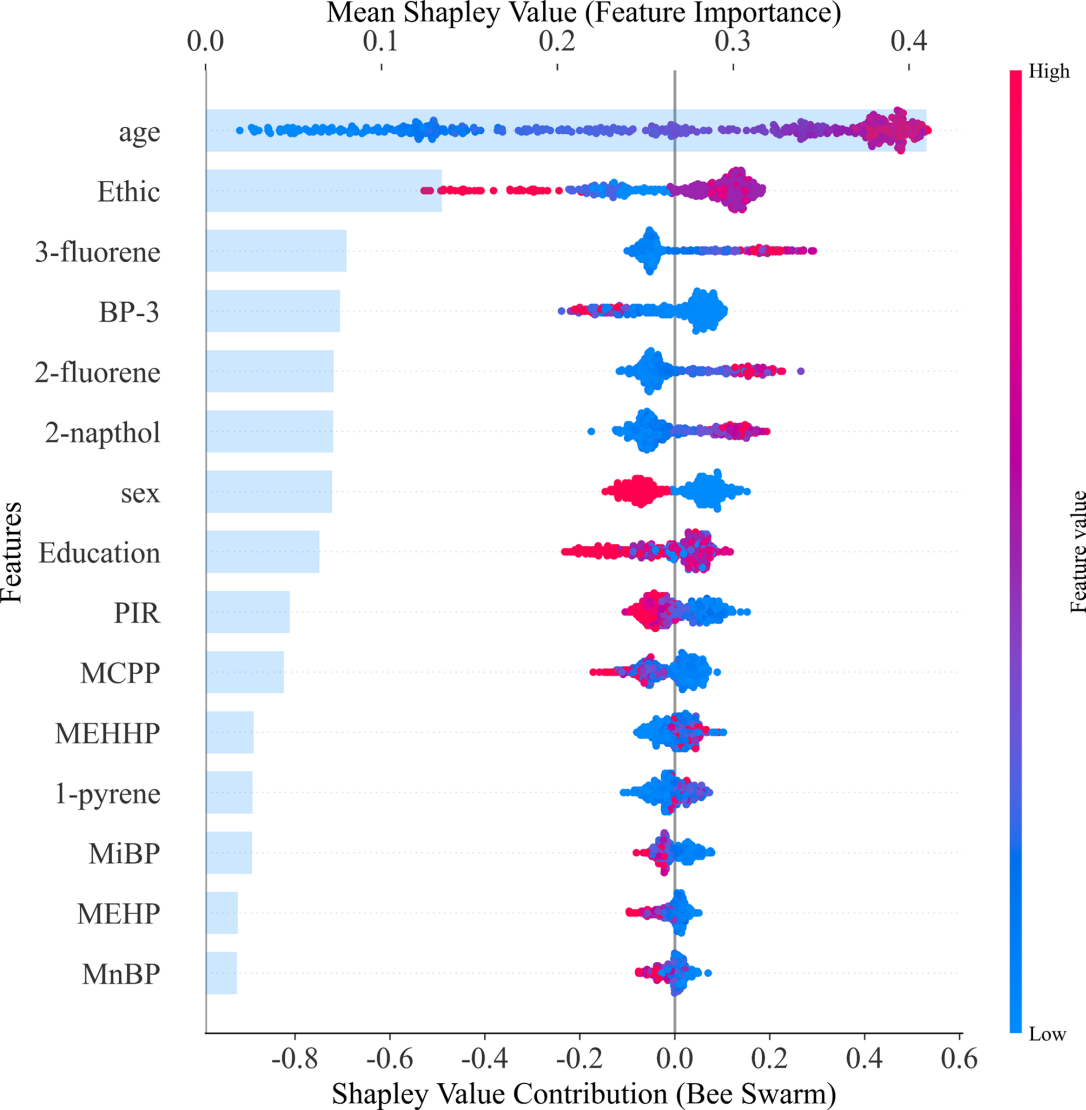
The SHAP summary plot

To enhance the interpretability of the prediction model, SHAP values were employed to quantify the contribution of each feature to cardiovascular risk prediction (Fig. 6). The SHAP summary plot illustrates the importance of features in a hierarchical manner, with each point representing the contribution of a specific feature for an individual. Red and blue indicate high and low feature values, respectively. The results highlight that advanced age, female gender, higher body mass index, and lower education level are strongly associated with increased cardiovascular risk. Furthermore, socio-demographic factors such as lower education levels and racial differences (specifically non-Hispanic White) underscore the interaction between environmental and socio-economic determinants in cardiovascular health. These findings align with cohort studies that emphasize systemic inequality in risk stratification [33]. The alignment of SHAP values with epidemiological evidence strengthens the biological validity of the model and its relevance for risk mitigation strategies.

Additionally, the positive correlation between EDCs and cardiovascular risk is consistent with prior research linking these chemicals to oxidative stress and endothelial dysfunction—key pathways in cardiovascular disease [34]. Among the 18 EDCs selected from urine samples, there are notable differences in their contributions to the model. (PAHs such as 3-Hydroxyfluorene, 2-Hydroxyfluorene, and 2-Hydroxynapthol exhibit positive contributions to the model, while phenolic compounds like BP-3 and certain phthalate metabolites such as MCPP have a significant impact. Other phthalates, including MEHHP, MiBP, MEHP, and MnBP, show negative contributions. The positive contribution of PAHs like 3-Hydroxyfluorene and 2-Hydroxyfluorene to cardiovascular risk is significant, which mirrors the results of previous cohort studies [35]. PAHs are known teratogens, carcinogens, and mutagens, and have been linked to cardiovascular disease and immune dysfunction [36, 37]. BP-3 has also been shown to affect inflammatory biomarkers associated with cardiovascular disease [38], and animal studies indicate that chronic exposure may lead to myocardial hypertrophy and damage in zebrafish [39].

### Molecular docking

**Fig. 7:**
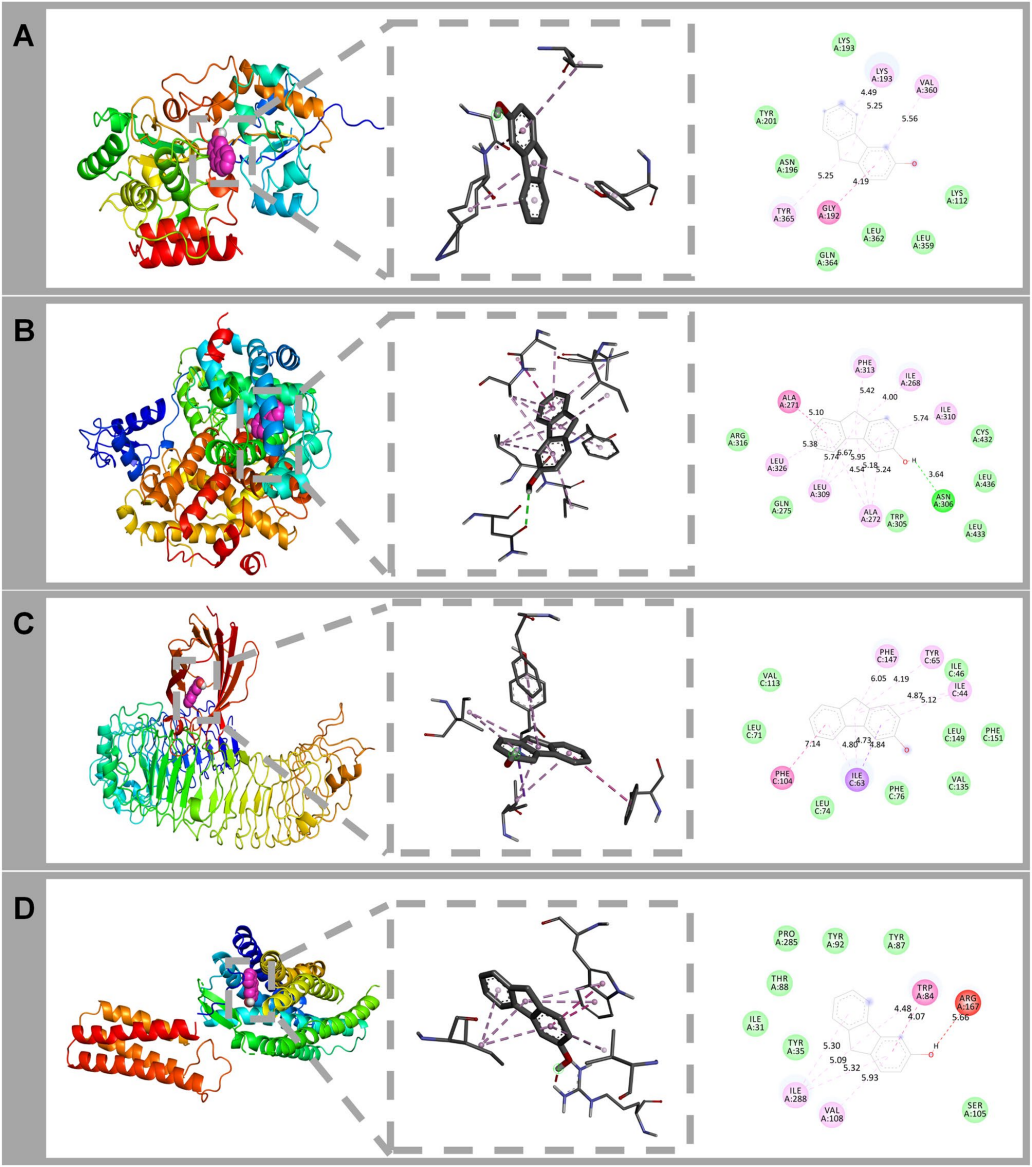
Binding interactions of 3-HF and four proteins: 3FXI(**A**), 2HEU(**B**), 3DZU(**C**), and 4YAY(**D**)

To further explore the relationship between EDCs and protein receptors, we selected 3-Hydroxyfluorene (3-HF) as a representative EDCs for docking analysis with relevant CVD - related protein targets. The binding patterns of 3-HF with these protein targets were identified (Fig. 7). We calculated the binding scores for the four targets, which were, in descending order, 2HEU (−9.4), 3DZU (−8.6), 3FXI (−8.4), and 4YAY (−7.8), with the lower ones being more stable. These binding energies suggest stable interactions. Based on these results, we selected the most stable target, 2HEU, which had the lowest binding energy, for further analysis [40] (Fig. 7 B).

The analysis revealed that key residues of the 3-HF-2HEU complex, such as LEU A: 326, ALA, ILE A: 31, and PHE, form hydrogen bonds and promote reactive oxygen species (ROS) generation through multimodal interactions. The LEU A: 326 residue may suppress the expression of inflammatory factors like interleukin-6, tumor necrosis factor-alpha, and monocyte chemoattractant protein-1 by inhibiting the mTOR signaling pathway, which could subsequently reduce triglyceride and low-density lipoprotein levels [41, 42]. ILE has been linked to increased thrombosis risk by enhancing the acetylation of pro-inflammatory protein-3 in platelets [43]. Meanwhile, the hydrophobic side chains of PHE contribute to tight packing, and an increase in phenylalanine leads to the breakdown of phenylalanine into toxic metabolites such as phenylpyruvic acid, phenyllactic acid, and phenylacetic acid. These metabolites promote oxidative stress, inhibit the oxidation rate of NAD-dependent substrates, and enhance ROS production [44]. Additionally, the amino group of ASN forms short hydrogen bonds with the hydroxyl group (−OH) of 3-HF, which directly interferes with the assembly interface of p22phox and p47phox complexes. This interaction may disrupt the maintenance of the reduced state of heme iron cofactors by blocking the SH3 domain of p47phox from binding to the proline-enriched region of p22phox, thus inhibiting the transmembrane transport of superoxide anions (O_2_^-^) released into the extracellular environment. This disruption leads to a reduction in ROS generation and endothelial-dependent vasodilation dysfunction. The binding of 3-HF may, therefore, inhibit receptor activation by altering the binding affinity of these receptors, interfering with the normal activation of the associated signaling pathways. This provides molecular evidence that environmental pollutants, such as EDCs, exacerbate cardiovascular disease by promoting redox imbalance.

### Molecular dynamics simulation

The NOX_2_-p22phox complex (2HEU) consists of a 22 kDa subunit (p22phox) and a glycosylated 91 kDa protein (gp91phox), also known as NOX_2_, forming the flavonoid cytochrome b558 (Vignais, 2002). The normal operation of this complex is closely related to cardiovascular health [45, 46].

#### Molecular dynamics trajectory analysis

**Fig. 8:**
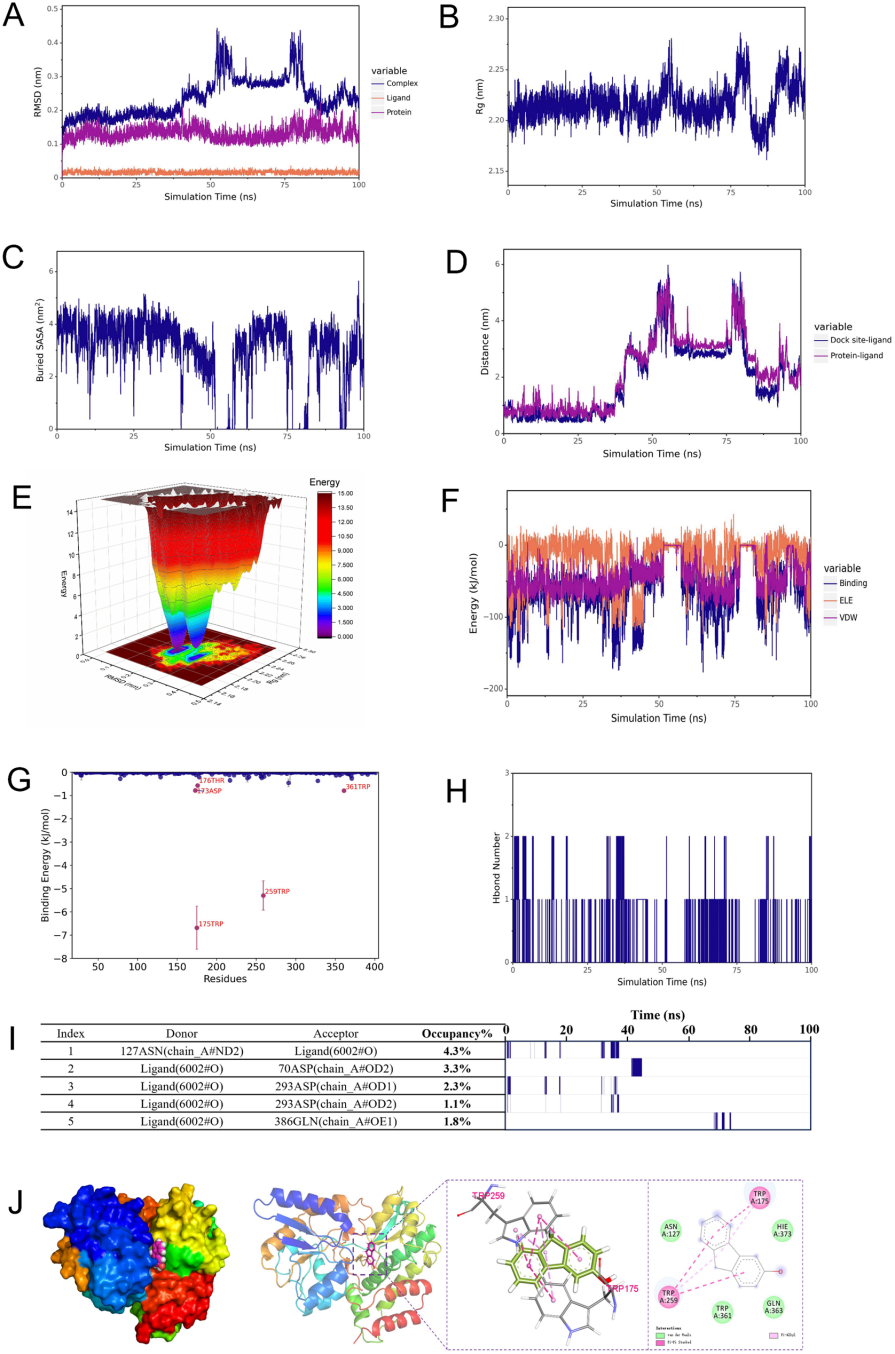
RMSD (**A**), R_g_ (**B**), buried SASA (**C**), DCOM (**D**), free energy landscape (**E**), VDW and ELE (**F**), the per-residue energy contribution spectrums (**G**), number of hydrogen bond (**H**), and hydrogen bond occupancy (**I**), structural and interaction analysis of conformations at stabilization (**J**); obtained from the MD simulation system between 3-HF and 2HEU

RMSD (Root Mean Square Deviation) is a measure of the similarity between two molecular conformations, which can help track changes in molecular structure relative to the initial structure during simulations. A lower RMSD indicates that the structures are more similar [47]. In this study, we analyzed the RMSD of the 2HEU-3-HF complex over a 100 ns molecular dynamics simulation. The RMSD of the complex showed a smooth state until 35 ns, with an average RMSD of the initial solvated complex being less than 0.3 Å, indicating stable binding (Fig. 8A). Furthermore, the RMSD of the complex was consistently higher than that of the free protein and ligand throughout the simulation, suggesting that the binding of 3-HF improved the stability of the complex.

When polycyclic aromatic hydrocarbons such as 3-HF bind stably to 2HEU, their function may be impacted through various mechanisms. First, the hydrophobic aromatic ring structure of 3-HF may occupy the electron transfer channels in the transmembrane helical region, creating steric hindrance that interferes with the efficiency of electron transfer from cytoplasmic NADPH/FAD to extracellular oxygen molecules. Previous experimental evidence suggests that this binding could lead to abnormal membrane localization and altered regulation of ROS generation in NOX2-p22phox complexes. However, further experimental verification is required to determine the precise effect on ROS generation rates. During the simulation, the detachment of 3-HF from the protein led to significant fluctuations in the RMSD after 35 ns. This dynamic instability in the binding mode may disrupt the transmembrane electron transfer function of NOX2-p22phox, reduce the efficiency of NADPH oxidase-generated ROS, and alleviate oxidative stress damage [48]. Therefore, in the subsequent analysis of structure and binding energy, the stable conformation before 35 ns will be mainly analyzed.

The radius of gyration (R_g_) is a measure of the distribution of atomic positions relative to the center of mass, providing insight into the overall structural compactness of molecules [49, 50]. Smaller R_g_ values indicate more compact molecules, while larger R_g_ values suggest looser, more flexible structures. In this study, we calculated the R_g_ of the 3-HF-2HEU complex. For the first 35 ns, the R_g_ remained stable, but it showed significant fluctuations after 35 ns due to the detachment of 3-HF from the protein. During the first 35 ns, the R_g_ of the complex gradually increased, then decreased, and eventually stabilized. This indicates that the complex initially loosened and then contracted after binding, ultimately reaching a tight, stable state (Fig. 8B). The observed compactness may be due to the hydrophobic interactions between the aromatic ring of 3-HF and the transmembrane region (ILE-310) [51], and transient hydrogen bonding between hydroxyl oxygen and ASN-306 [52]. These interactions help limit the spacing of the transmembrane helices and enhance the structural rigidity of the complex [53]. Once the complex stabilized, R_g_ remained constant, reflecting the tight coupling between the transmembrane region and the cytoplasmic domain [54]. However, after 35 ns, the significant fluctuation of R_g_ could be attributed to the detachment of 3-HF and conformational relaxation in the transmembrane region, leading to an expansion of the transmembrane helix spacing and increased structural heterogeneity [55]. This conformational extension weakened the tightness of the complex, while the restoration of the Cys-432 disulfide bond intensified the stretching of the transmembrane-cytoplasmic interface [56].

#### Stability analysis

Buried Solvent Accessible Surface Area (SASA) is a critical parameter used to assess the size of the binding interface between ligands and proteins. A larger SASA typically indicates stronger intermolecular interactions [57]. Any form of binding between small molecules and proteins can alter the SASA, potentially leading to structural rearrangement of the protein [58]. In our study, the buried SASA gradually stabilized before 35 ns, indicating that the binding region between 3-HF and 2HEU remained stable during this period (Fig. 8C). To further investigate the binding state of the protein-ligand complex, we analyzed the distance between the centroids of the initial binding site residues and the ligand centroids (DCOM) (Fig. 8D). The analysis showed that within the first 35 ns, the DCOM fluctuated slightly, but there was no significant distance fluctuation. This suggests that 3-HF and 2HEU did not detach during this period, and the small distance between them indicates stable binding.

The Free Energy Landscape (FEL) is a valuable tool in molecular simulation, offering a graphical representation of the distribution of free energy across different conformational states of a molecular system. It can display the relative stability of various possible conformations. In this study, we used the RMSD and R_g_ values of the complex as the free energy landscape for the 3-HF-2HEU complex (Fig. 8E). The FEL revealed two stable states. The first state corresponds to the tightly bound conformation of 3-HF and 2HEU, where the hydrophobic aromatic ring of 3-HF is stably embedded in the hydrophobic pocket of the transmembrane helical region. The second state corresponds to an intermediate conformation that arises after partial detachment of 3-HF, where the complex still maintains some structural stability, but the efficiency of electron transfer may decrease [59]. This bistable characteristic suggests that 3-HF may interact with 2HEU in a dynamic equilibrium, which is in line with the conformational regulation mechanisms of small molecule-protein complexes described in previous studies. This observation also underscores the cardiovascular system as the primary target site for inflammation induced by PAH exposure [60]. It is worth noting that the appearance of the second stable state may reflect the interference effect of 3-HF on the C-terminal membrane localization region of the p22phox subunit, leading to a change in the localization of the complex on the membrane [27, 45].

#### Analysis of the interaction between small molecules and proteins

Calculate the van der Waals forces and electrostatic interactions between complex small molecules and proteins without considering solvation, and analyze the changes in binding forces during the simulation process. Among them, VDW is the van der Waals force hydrophobic interaction, ELE is the electrostatic interaction, and Binding is the sum of VDW and ELE, which can represent the binding energy between small molecules and proteins without considering solvation effects. VDW and ELE in the composite have small fluctuations before 35 ns and remain stable (Fig. 8F). EIE is greater than VDW and fluctuates greatly, which determines the trend of Binding force. In the simulation, it was concluded that the binding energy gradually stabilized, indicating that the binding between small molecules and proteins gradually stabilized.

Considering the solvation energy and taking into account RMSD, R_g_, Distance, Buried SASA, and interaction energy, the stable state of the complex trajectory (0–35 ns) was selected and calculated using the MM-PBSA (Molecular Mechanics Poisson Boltzmann Surface Area) method to obtain the binding energy related energy (Table S1). ΔEele represents the electrostatic interaction between small molecules and proteins, ΔEvdw is the van der Waals interaction, ΔEpol is the polar solvation energy, which can represent electrostatic potential energy, ΔEnonpol is the non-polar solvation energy, which can represent hydrophobic interaction, ΔEMMPBSA is the sum of ΔEle, ΔEvdw, ΔEpol, and ΔEnonpol, and Gibbs Binding Energy binding energy ΔGbind is the sum ofΔ EMMPBSA and - T ΔS. Through the analysis of the table, van der Waals forces play a major role in the composition of the binding energy of the composite, hydrophobic interactions play a minor role, and electrostatic interactions play a supplementary role. In summary, 2HEU spontaneously binds to 3-HF through van der Waals forces as the dominant force during the binding process. The ΔEMMPBSA between small molecules and proteins is −39.445 ± 1.474 kJ/mol, indicating a high binding energy and affinity between the two.

Decompose the binding energy ΔEMMPBSA to obtain the contribution of each amino acid to the overall binding energy, evaluate the important amino acids in the protein, and identify the residues in the protein that contribute significantly to their respective binding energies as shown in the figure. From this figure, it can be seen that the key amino acid sites that bind small molecules in the protein include TRP-175, TRP-259, etc (Fig. 8G). These results indicate that the presence of hydrophobic amino acids may provide a certain hydrophobic force through the binding of 3-HF and 2HEU.

Hydrogen bonding is an important interaction between proteins and ligands. Hydrogen bonding is related to electrostatic interactions and can reflect the strength of electrostatic interactions. The number of hydrogen bonds between small molecules and proteins is relatively small, fluctuating between 0 and 1 (Fig. 8H). Analyze the stability of amino acid residues and hydrogen bonds formed with ligands in proteins by studying the hydrogen bond occupancy rate (frequency of hydrogen bond formation) between ligands and proteins. On the left are the acceptors, donors, and occupancy rates of hydrogen bond pairs; On the right is the frequency of hydrogen bond formation, and the density of the lines represents the frequency of hydrogen bond formation (Fig. 8I). As shown in the figure, the hydrogen bond stability between small molecules and proteins is relatively low.

Finally, the conformation under simulated stability was selected to analyze its structure and interactions. As shown in the figure, TRP 175 and TRP-259 formed Pi-Pi Stacked and Pi Alkyl hydrophobic interactions with small molecules, while amino acids such as ASN-127 and GLN-363 formed van der Waals interactions with 3-HF (Fig. 8J).

The binding of 3-HF and 2HEU complex may increase the risk of cardiovascular disease through multiple mechanisms. Structural studies have shown that 3-HF may interfere with the normal function of the complex through the following pathways: firstly, its hydrophobic structure can specifically bind to the p22phox-NOX2 interaction interface, disrupting the efficiency of electron transfer from NADPH to oxygen molecules via FAD and heme [27, 45]. Secondly, the conformational changes of p22phox induced by 3-HF may affect the assembly process of the complex. These disturbances eventually lead to an increase in the level of oxidative stress, which is closely related to the pathological process of cardiovascular diseases such as atherosclerosis and hypertension. [48]. Of particular note is that 2HEU dysfunction has been confirmed to be associated with endothelial dysfunction and increased inflammatory response, which may be an important molecular mechanism for the increased risk of cardiovascular disease in 3-HF exposed populations [61–63].

## Limitations

The present study suffers from a number of methodological limitations: the reliance on NHANES biomonitoring is unable to capture chronic exposure dynamics, and studies on the effects of mixed exposure modes still need to be further explored. Despite cross-validation using more comprehensive molecular dynamics simulations, the atomic scale resolution of the heterogeneous effects in the NOX2-p22phox complex is limited by the lack of an advanced topological approach. Future work will integrate longitudinal data, residue-level interaction networks, and wet-lab validation to remedy these shortcomings.

## Conclusion

This study analyzed the association between 18 endocrine disruptors and cardiovascular disease risk through interpretable machine learning. The CatBoost model is the best model for identifying cardiovascular disease risk associations. 3-Hydroxyfluorescene is significantly associated with CVD, and molecular docking and molecular dynamics simulations have shown that it binds to the oxidative stress receptor NOX2-p22phox, interfering with the normal function of its residues and affecting disease development.

## Electronic supplementary material

Below is the link to the electronic supplementary material.

Supplementary Material 1

## Data Availability

Data will be made available on request.
