# Supplementary material for: Machine learning-driven prediction models and mechanistic insights into cardiovascular diseases: deciphering the environmental endocrine disruptors nexus
Source: J Transl Med. 2025 Nov 12;23:1272. doi: 10.1186/s12967-025-07223-6 (PMC12613883; doi:10.1186/s12967-025-07223-6)
Supplement: Supplementary file 1 — Supplementary Material 1 [file 12967_2025_7223_MOESM1_ESM.docx]

**Supplementary Materials for:**

**Machine Learning-Driven Prediction Models and Mechanistic Insights into Cardiovascular Diseases: Deciphering the Environmental Endocrine Disruptors Nexus**

Wen-Min Yu^1#,^ Yu-Peng Chen^2#^, An-Le Cheng^3^, Zi-Yi Zheng^3^, Jia-Wen Wang^2*^, Xiong-Bo Liu^3*^, Jing-Xuan Zhou^3*^

1. Department of Cardiology, Jintan Affiliated Hospital of Jiangsu University, Changzhou, China

2. Department of Urology, Fujian Provincial Hospital, Fuzhou University Affiliated Provincial Hospital, Shengli Clinical Medical College of Fujian Medical University, Fuzhou, China.

3. School of Urban Planning and Design, Peking University Shenzhen Graduate School, Shenzhen, China

# Co-first authors.

*Correspondence author:

Jia-Wen Wang: 1811210684@pku.edu.cn

Xiong-Bo Liu: lxb666@stu.pku.edu.cn

Jing-Xuan Zhou: zhjx@stu.pku.edu.cn

Table S1. The calculated (MM/GBSA) binding free energy (kJ/mol) of 2HEU-3HF complexes.

Figure S1. The Confusion Matrix of Models.

**Table S1.** The calculated (MM/GBSA) binding free energy (kJ/mol) of 2HEU-3HF complexes.

| Complex index | Δvdw | Δele | ΔPB | ΔSA | Δ_MMPBSA_ (MM+PB+SA) | -Tds | dG |
| --- | --- | --- | --- | --- | --- | --- | --- |
| 1 | -66.122±3.216 | -3.688±1.633 | 40.583±5.295 | -10.218±0.498 | -39.445±1.474 | 22.104±4.519 | -17.341±3.722 |

Note:

Vdw is van der Waals force; ele is electrostatic interaction; PB is polar solvation energy; SA is hydrophobic interaction; MM is the sum of vdw and ele; MMPBSA is the sum of MM, pb and sa; - TdS is the entropy effect; dG is the Gibbs binding free energy


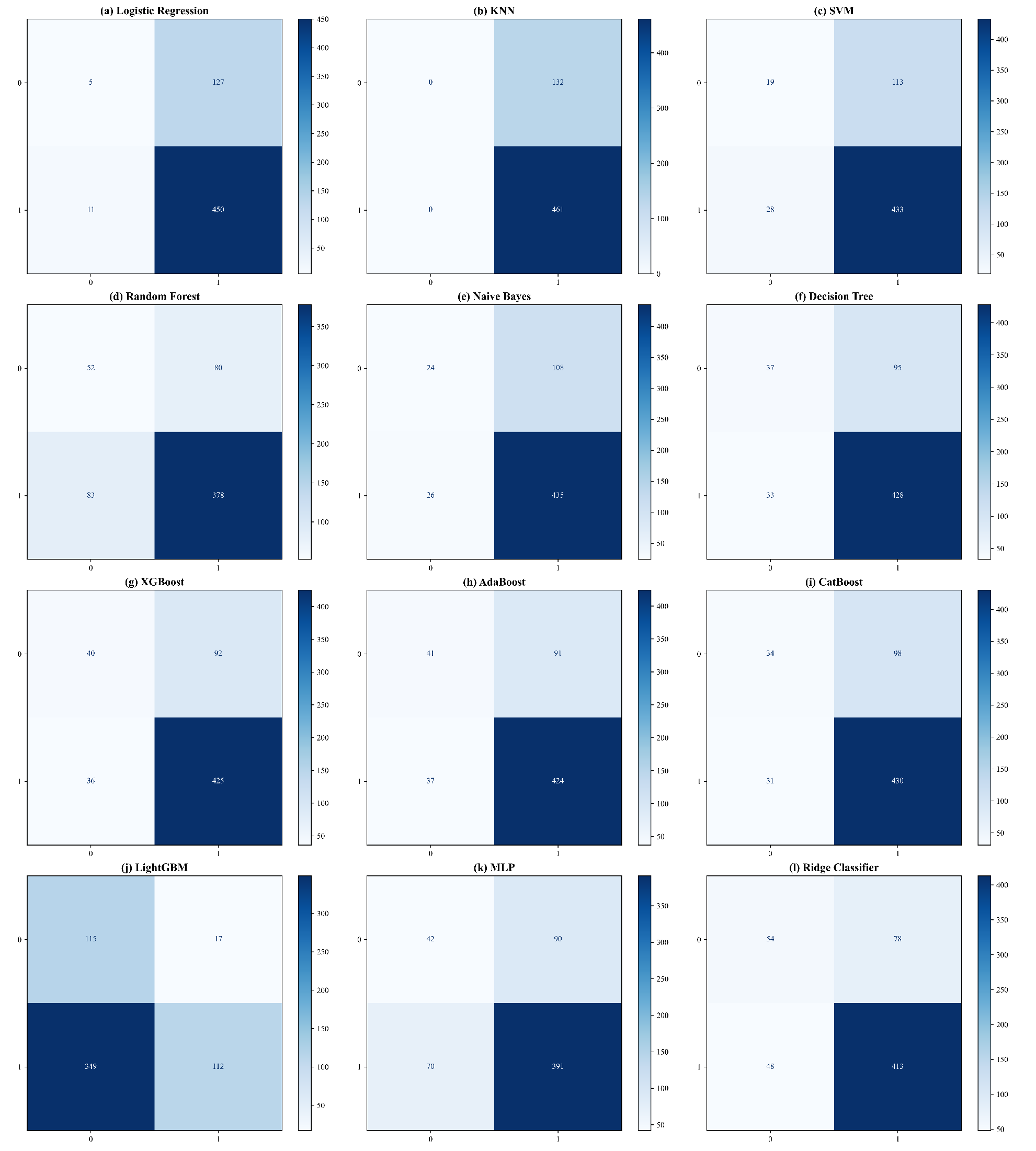


**Figure S1.** The Confusion Matrix of Model.
